# Enhanced cytotoxic effect of radiation and temozolomide in malignant glioma cells: targeting PI3K-AKT-mTOR signaling, HSP90 and histone deacetylases

**DOI:** 10.1186/1471-2407-14-17

**Published:** 2014-01-13

**Authors:** Eun Jung Choi, Bong Jun Cho, David J Lee, Yeo Hyeon Hwang, Sun Ha Chun, Hans H Kim, In Ah Kim

**Affiliations:** 1 Department of Radiation Oncology, Seoul National University Bundang Hospital, 166 Gumiro, Bundanggu, Seongnamsi Kyeonggido, South Korea; 2 Cancer Research Institute, Seoul National University 101 Daehak-ro, Jongno-gu, Seoul 110-779, South Korea

**Keywords:** Glioblastoma, Radiosensitization, Temozolomide, Pro-survival signaling

## Abstract

**Background:**

Despite aggressive treatment with radiation therapy and concurrent adjuvant temozolomide (TMZ), glioblastoma multiform (GBM) still has a dismal prognosis. We aimed to identify strategies to improve the therapeutic outcome of combined radiotherapy and TMZ in GBM by targeting pro-survival signaling from the epidermal growth factor receptor (EGFR).

**Methods:**

Glioma cell lines U251, T98G were used. Colony formation, DNA damage repair, mode of cell death, invasion, migration and vasculogenic mimicry as well as protein expression were determined.

**Results:**

U251 cells showing a low level of methyl guanine transferase (MGMT) were highly responsive to the radiosensitizing effect of TMZ compared to T98G cells having a high level of MGMT. Treatment with a dual inhibitor of Class I PI3K/mTOR, PI103; a HSP90 inhibitor, 17-DMAG; or a HDAC inhibitor, LBH589, further increased the cytotoxic effect of radiation therapy plus TMZ in U251 cells than in T98G cells. However, treatment with a mTOR inhibitor, rapamycin, did not discernibly potentiate the radiosensitizing effect of TMZ in either cell line. The mechanism of enhanced radiosensitizing effects of TMZ was multifactorial, involving impaired DNA damage repair, induction of autophagy or apoptosis, and reversion of EMT (epithelial-mesenchymal transition).

**Conclusions:**

Our results suggest possible strategies for counteracting the pro-survival signaling from EGFR to improve the therapeutic outcome of combined radiotherapy and TMZ for high-grade gliomas.

## Background

Glioblastoma multiforme (GBM) is the most common malignant primary brain tumor in adults and is among the most aggressive of all human tumors. Recent data from a randomized phase III clinical trial by the European Organization for Research and Treatment of Cancer/National Cancer Institute of Canada (EORTC 26981-22981/NCIC CE.3) suggest that concurrent and adjuvant temozolomide (TMZ) combined with radiation therapy results in significantly improved outcome in patients with GBM. However, despite this improvement the majority of patients with GBM relapse soon after treatment and the 2-year survival rate is only 26% [1].

Methylguanyl methyltransferease (MGMT) was the first molecular marker to serve as both a prognostic factor and a target for personalized therapy [2], and therapeutic resistance in MGMT-unmethylated tumors has emerged as an important clinical issue. Several other molecular biomarkers that regulate tumor growth, proliferation, and survival are being investigated as potential targets in the management of GBM. The Cancer Genome Atlas Research Network for GBM showed the role of ERBB2, NF1 and TP53, uncovers frequent mutations of the phosphatidylinositol-3-OH kinase regulatory subunit gene PIK3R1, and provides a network view of the pathways altered in the development of GBM [2]. One of the most common genetic alterations in primary GBM is over-expression of epidermal growth factor receptor (EGFR), which is observed in more than 50% of GBMs. Over-expression of EGFR and/or its constitutively activated variant EGFRvIII is associated with tumorigenesis and more aggressive phenotypes, such as, invasiveness and therapeutic resistance in GBM [3]. Preclinical data suggest that over-expression of EGFR confers radiation resistance on malignant glioma and that blocking EGFR restores radiosensitivity. However, the results of EGFR-targeted therapy trials for GBM, including gefitinib and erlotinib, have been disappointing due to diverse mechanisms of therapeutic resistance [4]. Emerging evidence indicates an important role that PTEN plays in predicting GBM response to EGFR-targeted therapy [5]. Aberrant PI3K/Akt/mTOR pathway has been shown to contribute to the resistant phenotype in glioma. Therefore, the EGFR/PI3K/Akt/mTOR pathway is regarded as the most amenable pathway to pharmacologic intervention in glioma [6]. We previously demonstrated an important role of PI3K-Akt-mTOR signaling in the radiation response [7]. In the present study, we evaluated the effect of targeting PI3K-Akt-mTOR signaling pathway, to identify effective strategies to improve therapeutic outcome when radiotherapy and TMZ are used concurrently to treat GBM.

The molecular chaperone HSP90 is known to stabilize Akt and oncogenic forms of mutant EGFR, both of which contribute to the growth of a variety of cancers including gliomas [8]. We previously reported that HDAC inhibitors potentiate radiation-induced cell killing in a panel of human cancer cells with activated EGFR signaling through diverse mechanisms [9]. A recent study also showed that HDAC inhibitors induced acetylation of HSP90, resulting in disruption of HSP90 chaperone function with EGFR and other oncogenic proteins in NSCLC [10]. Therefore, we also tested the effect of ligand-independent modulation using an HSP90 inhibitor and epigenetic modulation using a histone deacetylase (HDAC) inhibitor, focusing on targeting pro-survival signaling from EGFR. Additionally, the signaling cascades downstream of aberrant EGFR activation contribute to invasive phenotype in GBM and a mesenchymal feature of GBM is considered to be a major therapeutic obstacle for GBM treatment [11]. The recent recognition of mesenchymal change in glioblastoma and its association with more aggressive clinical phenotypes suggests that mechanisms that promote epithelial to mesenchymal transition (EMT) may be of great clinical relevance in GBM [12,13]. Thus, we also investigated inhibitory effects of these inhibitors in combination with TMZ on invasion, migration and vasculogenic mimicry formation of glioma cells.

## Methods

### Cell culture

The human GBM cell lines U251, U87, and T98G used in this study were obtained from the American Type Culture Collection (ATCC). All ATCC cell lines were authenticated by the company routine Cell Biology Program and were used within 6 months of receipt for this study. Cells were maintained and cultured according to standard techniques at 37°C in 5% (v/v) CO_2_ using culture medium recommended by the supplier. In all experiments, the different cell populations were first cultured in DMEM media containing 10% fetal bovine serum.

### Pharmacologic inhibitors

TMZ (Schering-Plough, Kenilworth, NJ, USA) was prepared by dissolving the drug in dimethyl sulfoxide (Sigma-Aldrich, St, Louis, MO, USA). PI103 (a pyridinylfuranopyrimidine inhibitor and a dual inhibitor of Class I PI3K and mTOR) and 17-Desmethoxy-17-N, N-dimethylaminoethylamino-geldanamycin, HCl, 17-N, N-Dimethylaminoethylamino-17-demethoxy-geldanamycin, HCl (17-DMAG), were obtained from Calbiochem® (Darmstadt, Germany). Rapamycin was obtained from Cell Signaling Technology, Inc (Beverly, MA, USA). Panobinostat (LBH589) was obtained from Selleck Chemicals LLC (Houston, TX, USA). Inhibitors were prepared as concentrated stock solutions in DMSO, stored at -20°C, and diluted in culture medium at the time of use. Control cells were treated with medium containing the same concentration of the drug carrier, DMSO.

### RNA interference

Two × 10^5^ cells were plated into each well of six well tissue culture plates. The next day (when the cells were 40–50% confluent), the culture medium was changed with antibiotics free medium. EGFR siRNA (5′- AAG AUC AUA AUU CCU CUG C -3′) was 19 nucleotides and nonspecific siRNA with similar GC content to the EGFR siRNA was used for control (Bioneer®, Daejeon, Korea).

Each EGFR siRNA and nonspecific control siRNA in reduced serum medium (OPTIMEM, Life Technologies) was transfected into cell using Lipofectamine 2000 (Invitrogen®, Carlsbad, CA) according to the manufacturer’s protocol. Forty-eight hours following transfection, cells were trypsinized, diluted to the appropriate cell density and plated in dishes for colony formation. Lysates from these cultures were screened for protein expression by Western blot analysis.

### Clonogenic assays

GBM cells were seeded into 6 well plates in 10% fetal bovine serum and on the first day of treatment the media were replaced with vehicle control or each drug with or without TMZ in DMEM media without fetal bovine serum. The media treated with drugs were replaced with DMEM media containing 10% fetal bovine serum after 24 hr. A specified number of cells were seeded into each well of 6-well culture plates. Cells were irradiated with 6MV X-ray from a linear accelerator (Clinac 6/100, Varian Medical Systems, Palo Alto, CA) at a dose rate of 2.46 Gy/min. As indicated, prior to irradiation cells were treated with TMZ (25 μM) with or without the inhibitors PI103 (0.4 μM), rapamycin (100 nM), 17-DMAG (25 nM), and LBH589 (20 nM) followed by incubation at 37°C for 10 to 14 days. Colonies were fixed with methanol and stained with 0.5% crystal violet; the number of colonies containing at least 50 cells was determined and surviving fraction was calculated. Radiation survival data were fitted to a linear-quadratic model using Kaleidagraph version 3.51 (Synergy Software, Reading, PA). We performed three independent experiments and each point on the survival curves represents the mean surviving fraction from triplicates. Sensitizer enhancement ratio (SER) was calculated as the ratio of the isoeffective dose at surviving fraction 0.5 and surviving fraction 0.05 in the absence of each inhibitor to that in the presence of each inhibitor.

### Western blot analysis

Cells were washed, scraped, and resuspended in lysis buffer (iNtRON Biotechnology, Seoul, Korea). Proteins were solubilized by sonication and equal amounts of protein were separated by SDS-PAGE and electroblotted onto polyvinylidene difluoride membranes (Millipore Corp., Bedford, MA, USA). Membranes were blocked in PBS containing 0.1% Tween 20 and 5% powdered milk and probed with primary antibody directed against p-EGFR (Tyr1068), p-Akt (Ser473), p-ERK (Tyr202/204), p-p70S6K (Thr421/Ser424), HSP70, HSP90, DNA-PKs (Thr2609), Rad51, caspase-3, LC3, MMP-2, E-cadherin, and EphA2 (Cell Signaling Technology, Inc.) at 1:1000 dilutions. Primary antibodies against MGMT (Abcam, Cambridge, UK) and Acetyl Histone H3 (Millipore Corp.) were used at a dilution of 1:1000. Antibodies against VEGF and β-actin (Santa Cruz Biotechnology, Santa Cruz, CA, USA) were used at dilutions of 1:500 and 1:5000, respectively. Membranes were washed and incubated with peroxidase-conjugated goat anti-rabbit or anti-mouse IgG secondary antibody (Jackson ImmunoResearch Laboratories, West Grove, PA, USA) at a dilution of 1:5000.

### Immunocytochemistry

Cells were seeded on chamber slides. At specified times after treatment, cells were fixed in 4% paraformaldehyde, and permeablized in methanol for 20 min. Cells were subsequently washed and blocked in PBS containing 2% bovine serum albumin for 1 h. Primary antibody against γH2AX (Cell Signaling Technology) was applied to the cells and incubated overnight. Secondary FITC anti-rabbit antibody (Molecular Probes, Eugene, OR, USA) was applied and incubated for 2 h. DAPI nuclear counter stain was applied at 1 μg/mL for 5 min. Slides were examined on an Axio Scope.A1 Imager fluorescent microscope. Images were captured and acquired using AxioCam MRc5 and acquisition software AxioVision v.4.4 (Carl Zeiss, Gottingen, Germany).

### Caspase-3/7 assay

Cells (3 × 10^4^ per well) were seeded in a 96-well plate with 200 μl culture medium. Cells were treated with TMZ with or without each inhibitor prior to irradiation. Casapse-3/7 activity was measured as per manufacturer’s instructions (Invitrogen).

### Annexin V-FITC/Propidium Iodide (PI) double-staining

Apoptosis was demonstrated using Annexin V-FITC/Propidium Iodide (PI) double-staining. Cells were seeded in 8-well chamber slides, treated with each inhibitor with or without TMZ prior to irradiation and double-stained with Annexin V-FITC and propidium iodide according to the manufacturer’s instruction (BD) and then analyzed under a fluorescence microscope (Carl Zeiss).

### Cellular senescence-associated β-galactosidase assay

Cellular senescence was examined by detecting the activity of β-galactosidase. Tumor cells were seeded in 8-well chamber slides, treated with reagents prior to irradiation, and then stained using Senescence β-Galactosidase Staining Kit (Cell Signaling Technology) according to the manufacturer’s instruction. Cells were examined using a light microscope.

### Modified Boyden chamber assay

Cell invasion was measured using a Transwell system (Corning, Rochester, NY, USA) that allows cells migrate through 8-μm pores in polycarbonate membranes. Inserts containing cells were placed in 24-well plates (Corning) in starvation medium. Cells were trypsinized and resuspended, and an aliquot of 10^4^ cells was added to the upper chamber. After 24 h, inserts were fixed in methanol and stained with 1% crystal violet.

### Wound healing assay

Cells were grown to confluence in 6-well plates (SonicSeal Slide; Nalge Nunc, Rochester, NY, USA) and then starved as described above. Each well was divided into a 2 × 3 grid. Using a 1-mL pipette tip, a line was scratched in each hemisphere of the well to wound the cells and the medium was replaced with starvation medium. Images were taken of the intersections of the linear cell wound and each grid line. The pictures of same area were taken immediately after a wound was inflicted to the cell and at time point 24 hrs. Migration rate was estimated from the distance that the cells moved, as determined microscopically. The distances between the edges of the wound were measured by using Image J software. The sixty measurements were taken for each experimental condition. The degree of mobility is expressed as percent of wound closure as compared with the zero time point. Migration rates were calculated using the following equation: (initial distance-final distance/initial distance) × 100.

### Vasculogenic mimicry formation assay

Vasculogenic mimicry (VM) formation assay was performed using a commercialized Matrigel assay kit (BD Biosciences, France). 200 μL ECM Matrigel was dropped in 48-well tissue culture plates and then incubated at 37°C for 2 hr. Cells were treated with TMZ (25 μM) with or without the inhibitors PI103 (0.4 μM), rapamycin (100 nM), 17-DMAG (25 nM), and LBH589 (20 nM) and then seeded onto the coated plate. After growth for 24 hr on the plate, VM formation was assessed using an inverted microscope.

### Statistical analysis

These results are expressed as the mean ± SD of three independent experiments. Data from these experiments were analyzed by Student’s *t* test (SPSS12.0 software). Significant differences were established at *P* < 0.05.

## Results

### Specific inhibition of EGFR using RNA interference

**Figure 1:**
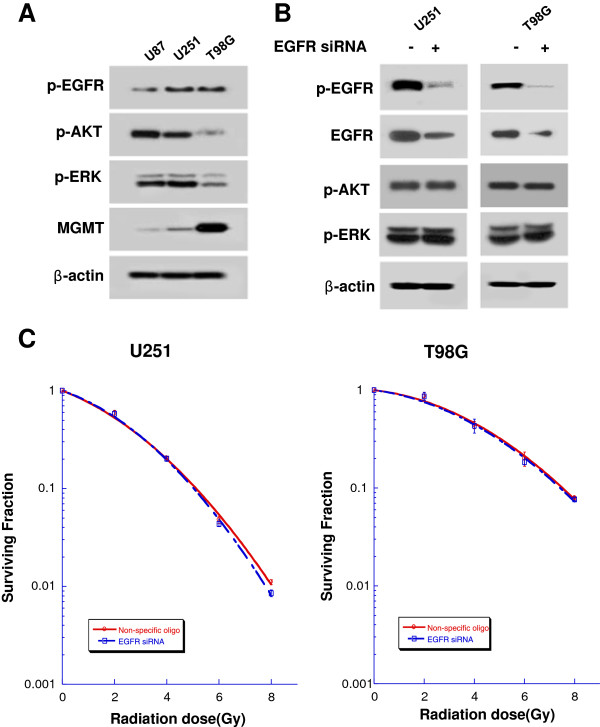
**Specific inhibition of EGFR does not result in radiosensitization of U251 and T98G cells. (A)** Forty-eight hours after serum starvation, western blot analysis showed low levels of MGMT expression in U87 and U251 cells, and a high level of MGMT expression in T98G cells. U251 and T98G showed similar levels of p-EGFR expression. **(B)** Western blot analysis of U251 and T98G cells transfected with EGFR-specific or nonspecific siRNA. **(C)** Cells were plated for colony formation assay 48 h after transfection with EGFR-specific or nonspecific siRNA and irradiated as indicated. Points on survival curves represent mean surviving fractions from minimum three experiments performed in triplicate.

First, we evaluated p-EGFR, MGMT expression levels in a panel of glioma cell lines. U251 and T98G showed similar levels of p-EGFR expression. U251 and U87 cells showed low level of MGMT, as previously described [14] which might highlight a high level of MGMT promotor methylation, compared with T98G (Figure 1A). To determine the effect of targeting EGFR signaling during the radiation response, U251 cells and T98G cells were transfected with either EGFR-specific siRNA or nonspecific siRNA. Specific inhibition of EGFR did not attenuate signaling through downstream mechanisms such as p-Akt, p-ERK (Figure 1B), and did not result in significant radiosensitization (sensitizer enhancement ratio at surviving fraction of 0.5 [SER0.5], 1.0) (Figure 1C).

### Targeting PI3K-Akt-mTOR pathway

**Figure 2:**
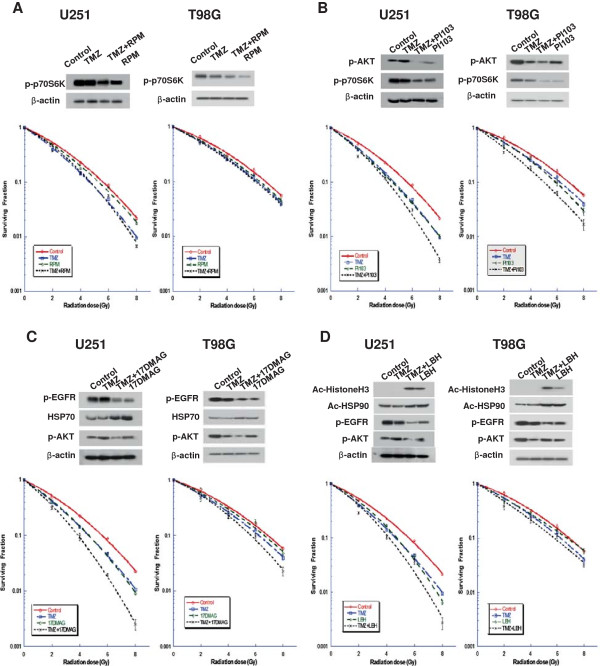
**Targeting PI3K-Akt-mTOR signaling. (A)** U251 and T98G cells were pretreated with rapamycin (RPM) plus TMZ for 24 h and subjected to western blot analysis using the indicated antibodies. Pretreatment with rapamycin (100 nM) plus TMZ (25 μM) did not have a synergistic radiosensitizing effect compared to TMZ alone treatment on U251 and T98G cells. **(B)** U251 and T98G cells were pretreated with a dual inhibitor of class I PI3K and mTOR signaling, PI103 (0.4 μM), and TMZ (25 μM) for 24 h. PI103 effectively enhanced the radiosensitizing effect of TMZ in both U251 and T98G cells. **(C)** U251 and T98G cells were pretreated with the HSP90 inhibitor 17-DMAG (25nM) and TMZ (25 μM) for 24 h. 17-DMAG enhanced the radiosensitizing effect of TMZ in U251 and T98G cells. **(D)** U251 and T98G cells were pretreated with TMZ (25 μM) and LBH589 (20 nM) for 24 h. LBH589 effectively potentiated the radiosensitizing effect of TMZ. Points on survival curves represent mean surviving fractions from minimum three experiments performed in triplicate.

We tried to determine whether inhibition of these targets would further increase the radiosensitizing effect of TMZ. Since inhibition of mTOR is a way to avoid possible side effects associated with inhibition of PI3K-Akt, we tested whether rapamycin would cause radiosensitivity in glioma cells. Pretreatment with rapamycin (0.1 μM) caused a dramatic reduction in the level of p-p70S6K, but did not discernibly potentiate the radiosensitizing effect of TMZ in either cell line (p > 0.05 for U251 and T98 G Cells, Figure 2A). As shown in Figure 2B, PI103, a dual inhibitor of class I PI3K and mTOR, markedly reduced p-Akt and p-p70S6K protein levels, and effectively potentiated the radiosensitizing effect of TMZ in both cell lines (p < 0.05 for U251 and T98G cells). Similar results were seen with U87 cells (Additional file 1: Figure S1A). Additional file 1: Tables S1 and Additional file 1: Table S2 show the sensitizer enhancement ratio (SER) for each inhibitor alone and combined with TMZ in U251, T98G, and U87 cells. PTEN-mutant U251 cells showed higher radiosensitizing effect of PI103 than that of T98G which has PTEN-wild type (SER_0.5_ 1.41 vs. 1.26).

### Ligand-independent modulation using HSP90 inhibitor

As shown in Figure 2C, pretreatment with a HSP90 inhibitor, 17-DMAG (25 nM), increased expression of HSP70 and attenuated levels of its client proteins, p-EGFR and p-Akt. 17-DMAG effectively potentiated the radiosensitizing effect of TMZ (p < 0.05 for U251 cells). This effect was more pronounced in U251 cells than in T98G cells at the higher radiation doses (Additional file 1: Tables S1 and Additional file 1: Table S2). Similar results were seen with U87 cells (Additional file 1: Figure S1B).

### Epigenetic modulation using HDAC inhibitor

As shown in Figure 2D, pretreatment with a HDAC inhibitor, LBH589 (20 nM), induced acetylation of histone H3, leading to acetylation of HSP90 and down-regulation of its client proteins p-EGFR and p-Akt. LBH589 effectively potentiated the radiosensitizing effect of TMZ (p < 0.05 for U251 cells). This effect was more pronounced in U251 cells than in T98G cells and occurred at higher radiation doses (Additional file 1: Tables S1 and Additional file 1: Table S2).

### Impairment of DNA damage repair following irradiation

**Figure 3:**
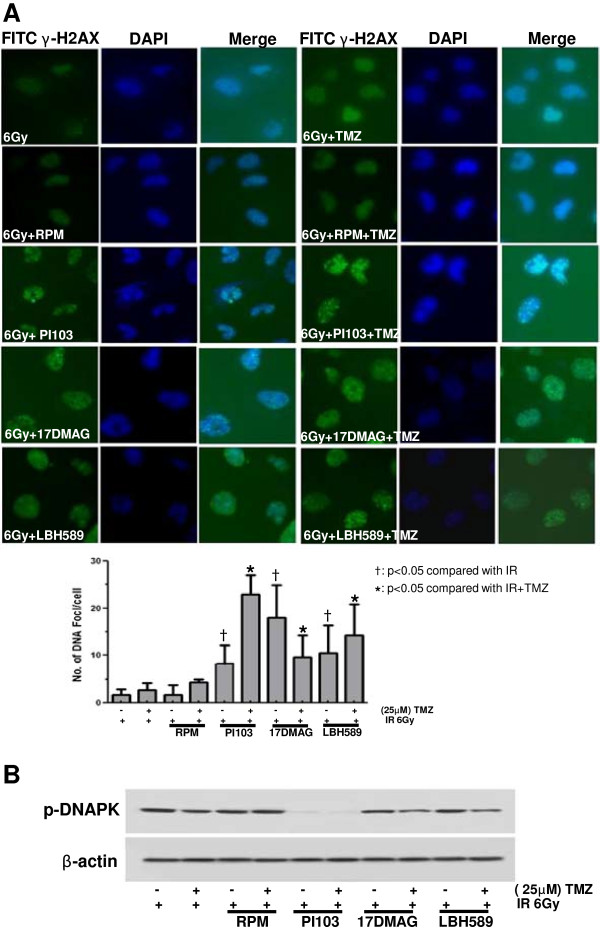
**Impairment of DNA damage repair following irradiation. (A)** U251 cells were pretreated with the indicated inhibitors plus TMZ before assessment of γH2AX foci formation. Mock-treated control cells were analyzed 6 h after irradiation with 6 Gy. Pretreatment of U251 cells with the dual inhibitor PI103, the HSP90 inhibitor 17-DMAG, or the HDAC inhibitor LBH589 plus TMZ caused marked prolongation of radiation-induced γH2AX foci formation 6 h after 6Gy irradiation. **(B)** Pretreatment of U251 cells with TMZ combined with PI103, 17-DMAG, or LBH589 attenuated p-DNA-PK expression.

U251 cells were pretreated with the indicated inhibitors plus TMZ before assessment of γH2AX foci formation. Mock-treated control cells were analyzed 6 h after irradiation with 6 Gy. Pretreatment of U251 cells with the dual inhibitor PI103, the HSP90 inhibitor 17-DMAG, or the HDAC inhibitor LBH589 combined with TMZ caused marked prolongation of radiation-induced γH2AX foci formation 6 h after irradiation with 6 Gy (Figure 3A), indicating delayed DNA damage repair. Pretreatment of U251 with PI103, 17-DMAG, or LBH589 combined with TMZ attenuated expression of p-DNA-PK (Figure 3B).

### Mode of cell death

**Figure 4:**
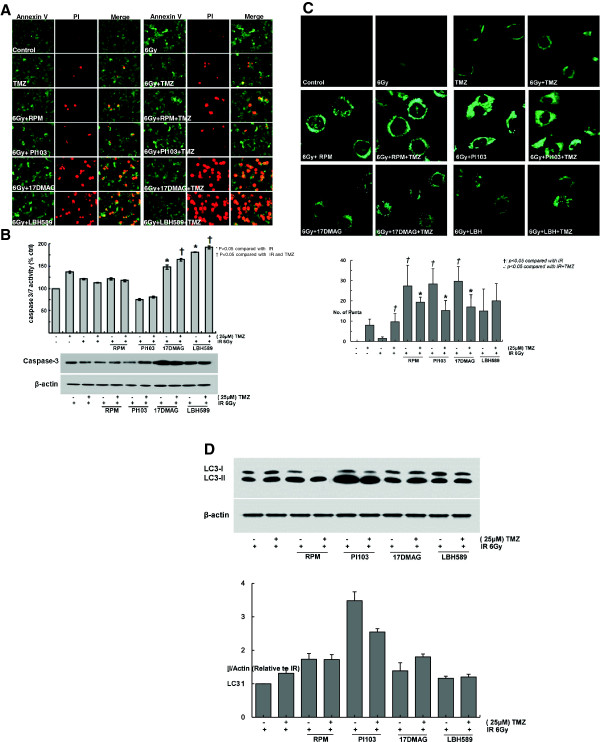
**Mode of cell death. (A)** Annexin-V-FITC/PI double staining images of each treatment at 24 h. Annexin-V-FITC staining targets the membranes of apoptotic cells, showing green fluorescence, while PI staining targets the nuclei of apoptotic cells, showing red fluorescence. The combination treatment of TMZ with 17-DMAG or LBH589 increased green and red fluorescence on U251 cells. **(B)** The combination treatment of TMZ with 17-DMAG or LBH589 increased caspase-3/7 activity and cleaved caspase3 expression within 24 h after combination treatment on U251 cells. Data are presented as the mean ± SD of three experiments. *P < 0.05 versus IR*.***†***P < 0.05 versus* IR and TMZ*.***(C)** Pretreatment of U251 cells with TMZ combined with rapamycin or PI103 increased punctate fluorescence or lysosomal localization of LysoTracker at 24 h. **(D)** Treatment with rapamycin or PI103 with or without TMZ increased LC3–II (16 kDa) expression in U251 cells at 24 h after each combined treatment. Each experiment was repeated three times with similar results.

Annexin V-FITC/PI double staining and Caspase 3/7 assay method were employed to examine apoptotic cell death. Annexin-V-FITC staining targets the membranes of apoptotic cells, showing green fluorescence, while PI staining targets the nuclei of apoptotic cells, showing red fluorescence. As shown in Figure 4A, the combined treatment of TMZ with 17-DMAG or LBH589 showed fluorescent green cell membranes and fluorescent red nuclei. Additionally, treatment of TMZ with 17-DMAG or LBH589 increased cleaved caspase3 expression and caspase-3/7 activity within 24 h after combination treatment on U251 cells (Figure 4B, P < 0.05). Pretreatment with TMZ combined with rapamycin or PI103 increased punctate fluorescence or lysosomal localization of LysoTracker in U251 cells at 24 h (Figure 4C). To further elucidate the mechanism underlying autophagy in U251 cells, we examined the effect of the combination treatment of each inhibitor with or without TMZ on the conversion of microtubule-associated protein light chain (LC3). Treatment with rapamycin or PI103 in the presence or absence of TMZ increased LC3–II (16 kDa) expression in U251 cells at 24 h after each combined treatment (Figure 4D). Senescence was examined by detecting the activity of β-galactosidase and no discernable change was detected in U251 cultures within 7 days after each treatment (Additional file 1: Figure S2).

### The effect on invasion, migration and vasculogenic mimicry of glioma cells

**Figure 5:**
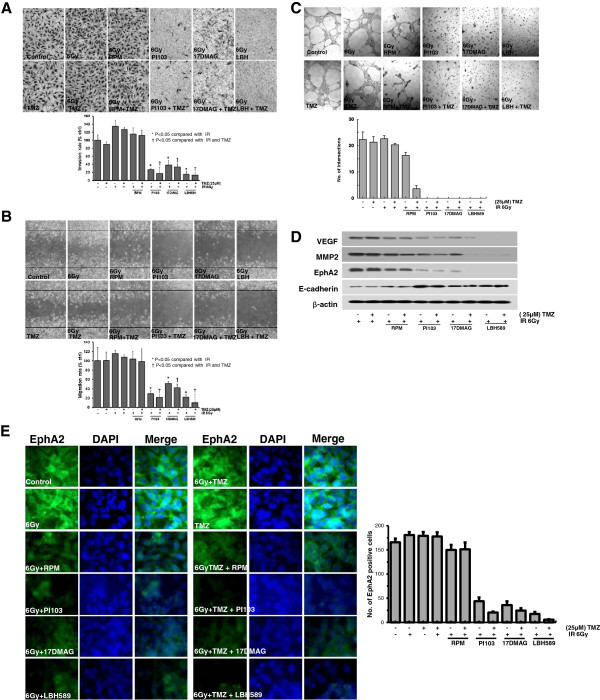
**Invasion, migration and vasculogenic mimicry formation of U251 glioma cells.** The effects of each treatment on invasion, migration, and vasculogenic mimicry (VM). **(A & B)** U251 cells invasion/migration was determined by using Modified Boyden chamber assay and Wound healing assay. Stained cells in representative fields (100×). The number of cells that had invaded and migrated 24 hours after treatment shown as histogram. Data are presented as the mean ± SD of three experiments. *P < 0.05 versus IR*.***†***P < 0.05 versus* IR and TMZ*.***(C)** The ability of U251 cells to form VM when plated on matrigel was determined in each treatment. Photographs of representative VM formation fields are shown (200×). **(D)** The combination treatment of TMZ with PI103 or 17-DMAG or LBH589 resulted in down-regulation of VEGF, MMP-2, and EphA2 expression and up-regulation of E-cadherin expression, by Western blot analysis. β-actin was detected as loading control. **(E)** The level of EphA2 immunofluorescence is visibly lower in the combination treatment of TMZ with PI103 or 17-DMAG or LBH589 compared to TMZ alone treatment in U251 cells. Each experiment was repeated three times with similar results.

Invasion, and migration are key processes of tumor progression and are tightly linked to tumor recurrence and therapeutic resistance in glioblastoma [8]. Radiation (6 Gy) and/or TMZ treatment did not cause the inhibition of migration and invasion in U251 cells. However, the combination treatment of TMZ with PI103 or 17-DMAG or LBH589 markedly inhibited the ability of migration and invasion of U251 glioma cells (Figure 5A, B, P < 0.05).

Vasculogenic mimicry (VM) is known as non-endothelial tumor cell-lined microvascular channels in aggressive tumors and is associated with aggressive and invasive nature of gliomas [13]. Since VM has a totally different structure from endothelium-dependent vessels, traditional anti-vascular therapies aiming at endothelial cells have no remarkable effects on malignant tumor with VM [15]. To evaluate the inhibitory effect of each treatment on VM, we performed VM formation assay using U251 glioma cells. PI103 or 17-DMAG or LBH589 combined with radiation and/or TMZ significantly impaired VM formation of U251 glioma cells compared with TMZ alone treatment (Figure 5C).

Consistent with the reduction of invasion, migration and VM formation, the combination treatment of TMZ with PI103 or 17-DMAG or LBH589 showed a decrease in expression of vascular endothelial growth factor (VEGF), matrix metalloproteinase (MMP) 2 and EphA2. In contrast, the treatment of TMZ with PI103 or 17-DMAG or LBH589 led up-regulation of epithelial marker E-cadherin (Figure 5D). As shown in Figure 5E, abundant staining for EphA2 was observed in control, TMZ, and rapamycin with or without TMZ. In contrast, the level of EphA2 was considerably lower when the cells were treated by TMZ with PI103 or 17-DMAG or LBH589.

## Discussion

The current standard of care for malignant glioma is initial treatment with radiation therapy combined with TMZ; however, malignant gliomas usually recur with a median time to progression of approximately 7 months [1]. Two decades of molecular studies have identified important genetic events such as dysregulation of growth factor signaling via amplification or mutation of receptor tyrosine kinase genes; activation of PI3K pathway; and inactivation of p53 and Rb tumor suppressor pathways [2]. In this study, we tried to identify the potential targets for counteracting the pro-survival signaling implicated in radioresistance of malignant glioma cells and to get insight into potential strategies to improve the therapeutic outcome of radiotherapy and TMZ in the management of GBM.

Inhibition of signal transduction pathways may provide the basis for a new paradigm of GBM therapy, based on the fact that most human gliomas exhibit aberrant activation of a pro-survival/pro-growth signaling network. EGFR is one of the most attractive therapeutic targets in GBM since the gene is amplified and over-expressed in approximately 40% of primary GBMs, especially those of the classical subtype. Nearly half of tumors with EGFR amplification also express a constitutively active EGFR mutant, EGF variant VIII (EGFRvIII), which has an in-frame deletion of exons 2–7 within the EGFR extracellular domain [16,17]. Clinical trials with EGFR kinase inhibitors such as gefitinib and erlotinib did not show a significant benefit on overall survival or progression-free survival in patients with malignant glioma [4]. Given the role of this growth factor receptor in gliomagenesis [18], the failure of EGFR inhibitors in GBM patients was particularly disappointing. Understanding the molecular mechanism of resistance may provide insight into the development of alternative strategies to tackle this issue.

Some studies found that tumors with EGFRvIII [7] and intact PTEN and tumors with low p-Akt levels are more likely to respond to EGFR inhibitors [19]. Several investigators have identified loss of the PTEN tumor suppressor as a resistance factor for EGFR kinase inhibitor therapy [5,20,21]. Vivanco et al. also showed a critical role of PTEN in downregulation of activated EGFR. The PI3K/Akt/mTOR pathway is a critical regulator of tumor cell metabolism, growth, proliferation, and survival. In malignant gliomas, activity of this signaling network is frequently increased because of receptor tyrosine kinase over-activity, loss of PTEN tumor suppressor, and/or mutated oncogenic PI3K subunits [6]. We observed a PTEN-mutant gloma cells showed higher radiosensitizing effect of PI103 than that of PTEN-wild type glioma cells. Our finding also supports the potential and rationale for PI3K targeting strategy in the treatment of malignant glioma having PTEN loss.

Attempts to inhibit the PI3K pathway with pan-PI3K inhibitors such as LY294002 have not progressed to clinical use due to concerns over organ toxicity and a lack of selectivity [22,23]. Inhibition of the pathway using rapamycin resulted in paradoxical activation of Akt through loss of negative feedback in a subset of patients, which in turn was related to shorter time-to-progression during postsurgical maintenance rapamycin therapy [24]. The limited single-agent activity of rapamycin analogs in several GBM trials [25,26] provides a rationale for ongoing clinical trials with dual PI3K/mTOR inhibitors in GBM. A clinical trial of a dual PI3K/m-TOR inhibitor, XL765, in combination with TMZ is currently underway for GBM [27]. Our results are in line with previous reports since combined treatment with TMZ and a dual PI3K/m-TOR inhibitor, XL765, has been successfully tested in glioma cell lines [23,27]. Although rapamycin was a strong inducer of autophagy, it did not increased cytotoxicity of radiation therpy combined with temozolomide. In contrast, PI103 which is a dual inhibitor of class I PI3K and m-TOR prolonged gammH2AX foci formation with downregulation of p-DNA-PK, increased autophagy and increased cytotoxicity of radiation and temozolomide. We speculated that the impairment of DNA damage repair following radiation is potential mechanism of radiosensitization seen with this compound. Based on our results, we propose dual targeting PI3K/m-TOR with PI103 as a viable therapeutic strategy which should be explored to bypass the therapeutic resistance of GBM.

The data from TCGA for GBM indicate that tumorigenesis and progression involve multiple molecular abnormalities [28]. HSP90, a molecular chaperon, is essential for the stability and function of many oncogenic client proteins that are frequently dysregulated in GBM, such as mutant EGFR, Akt, and p53. Since HSP90 is essential for the function of normal cells as well as tumor cells, one might be concerned that inhibition of its functions might not be selective for malignancy. Both preclinical and clinical observations, however, have shown that HSP90 inhibitors can be given in vivo at doses and schedules that exert antitumor activity without causing host toxicity [29]. In addition to counteracting pro-survival signaling, HSP90 inhibitors block cell motility and invasion by suppressing multiple pro-invasive and pro-angiogenic cellular processes, such as MMP-2, VEGF [30] and EphA2 activity [31]. Moreover, HSP90 can play a role in DSB repair and the activation of cell cycle check point [32]. Inhibition of multiple signaling circuitries through the abrogation of HSP90 may be an effective treatment strategy for highly recalcitrant tumors such as GBM [33-35].

HDAC inhibitors (HDIs) target epigenetic modifications that interfere with transcriptional regulation and can induce growth arrest and cell death [36-38]. We previously reported that HDIs potentiate radiation-induced cell killing in a panel of human cancer cells through diverse mechanism: LBH589 preferentially radiosensitized human glioma cells that exhibited activated EGFR signaling due to the EGFRVIII mutation. Treatment with LBH589 led to acetylation of HSP90, which induced down-regulation of the client oncoproteins EGFR and decreased levels of p-Akt [39]. LBH589 has also been reported to inhibit angiogenesis [40] and induce apoptosis, and delay of DNA damage repair in lung cancer cells with activated EGFR [41]. Srivastava et al. reported that MS-275 inhibited tumor cell proliferation, angiogenesis, metastasis and reversing EMT in vivo breast cancer xenograft model by causing “cadherin switch” and decreased expression of VEGF, HIF-1, MMP-2 and MMP-9 [42]. In our results, LBH589 significantly blocked migration, invasion, and vasculogenic mimicry formation through the down-regulation of VEGF, MMP-2, EphA2 and up-regulation of E-cadherin in U251 glioma cells. Given the link between mesenchymal feature and the progression of GBM, attacking EphA2, VEGF, and MMP-2 expression by these agents might be a potential strategy to improve the therapeutic outcome of combined radiotherapy and TMZ for GBM.

## Conclusions

Taken together, our data suggest that targeting PI3K and mTOR pathway, ligand-independent modulation using an inhibitor of HSP90, or epigenetic modulation through inhibition of HDAC could be potential strategies to improve the therapeutic outcome of GBM. Increased radiosensitizing effects of combination therapy with TMZ were associated with impairment of DNA damage repair, the induction of apoptosis or autophagy, and the reversion of EMT. Since the biology of malignant glioma involves a complex network of interconnected signaling pathways resulting in cell growth, survival and the invasive phenotype, careful preclinical interrogation is necessary to determine optimal treatment combinations.

## Abbreviations

EGFR: Epidermal growth factor receptor; EGFRvIII: EGF variant VIII; EMT: Epithelial to mesenchymal transition; GBM: Glioblastoma multiform; HDAC: Histone deacetylase; MGMT: Methylated methyl guanine transferase; TMZ: Temozolomide; VM: Vasculogenic mimicry.

## Competing interests

The authors declare that they have no competing interests.

## Authors’ contributions

IAK designed and supervised the study, was involved in data analyses and wrote the finalized manuscript. EJC and BJC performed most of initial laboratory work. DJL, SHC, and HHK were contributed to cell cultures and clonogenic assays. YHH and DJL performed additional experiments and helped the revision of this manuscript. All authors read and approved the final manuscript.

## Pre-publication history

The pre-publication history for this paper can be accessed here:

http://www.biomedcentral.com/1471-2407/14/17/prepub

## Supplementary Material

Additional file 1: Table S1 Sensitizer enhancement ratio of U251 cells. **Table S2.** Sensitizer enhancement ratio of T98G cells. **Table S3.** Sensitizer enhancement ratio of U87 cells. **Figure S1.** Clonogenic survival of U87 glioma cells after each treatment. (A) The effects of PI103 and TMZ on the radiosensitivity of U87MG. (B) The effects of 17DMAG and TMZ on the radiosensitivity of U87MG. (C) The effects of LBH589 and TMZ on the radiosensitivity of U87MG. Each experiment was repeated three times with similar results. **Figure S2.** Cellular Senescence-Associatedβ-Galactosidase Assay in U251 glioma cells after each treatment. **Figure S3.** Invasion, migration and vasculogenic mimicry formation of U251 glioma cells (without radiation). Click here for file
