# Supplementary material for: Enhanced cytotoxic effect of radiation and temozolomide in malignant glioma cells: targeting PI3K-AKT-mTOR signaling, HSP90 and histone deacetylases
Source: BMC Cancer. 2014 Jan 13;14:17. doi: 10.1186/1471-2407-14-17 (PMC3910677; doi:10.1186/1471-2407-14-17)

Supplementary Table

Supplementary Table 1. Sensitizer enhancement ratio of U251 cells

Inhibitor	SER $_{0.5}$	SER $_{0.05}$
RPM	1.06	1.04
TMZ	1.41	1.17
TMZ+RPM	**1.21**	**1.18**
PI103	1.41	1.20
TMZ	1.41	1.17
TMZ+PI103	**1.55**	**1.34**
17DMAG	1.31	1.18
TMZ	1.41	1.17
TMZ+17DMAG	**1.55**	**1.39**
LBH589	1.27	1.10
TMZ	1.41	1.17
TMZ+LBH589	**1.50**	**1.35**

Supplementary Table 2. Sensitizer enhancement ratio of T98G cells

Inhibitor	SER $_{0.5}$	SER $_{0.05}$
RPM	1.13	1.04
TMZ	1.26	1.07
TMZ+RPM	**1.23**	**1.10**
PI103	1.26	1.14
TMZ	1.26	1.07
TMZ+PI103	**1.65**	**1.30**
17DMAG	1.16	1.03
TMZ	1.26	1.07
TMZ+17DMAG	**1.29**	**1.20**
LBH589	1.24	1.01
TMZ	1.26	1.07
TMZ+LBH589	**1.40**	**1.14**

Supplementary Table 3. Sensitizer enhancement ratio of U87 cells

Inhibitor	SER $_{0.5}$	SER $_{0.05}$
PI103	1.05	1.08
TMZ	1.07	1.09
TMZ+PI103	1.12	1.21
17DMAG	1.02	1.03
TMZ	1.07	1.09
TMZ+17DMAG	1.08	1.20
LBH589	1.06	1.05
TMZ	1.07	1.09
TMZ+LBH589	1.2	1.20

Supplemental data Fig.S1

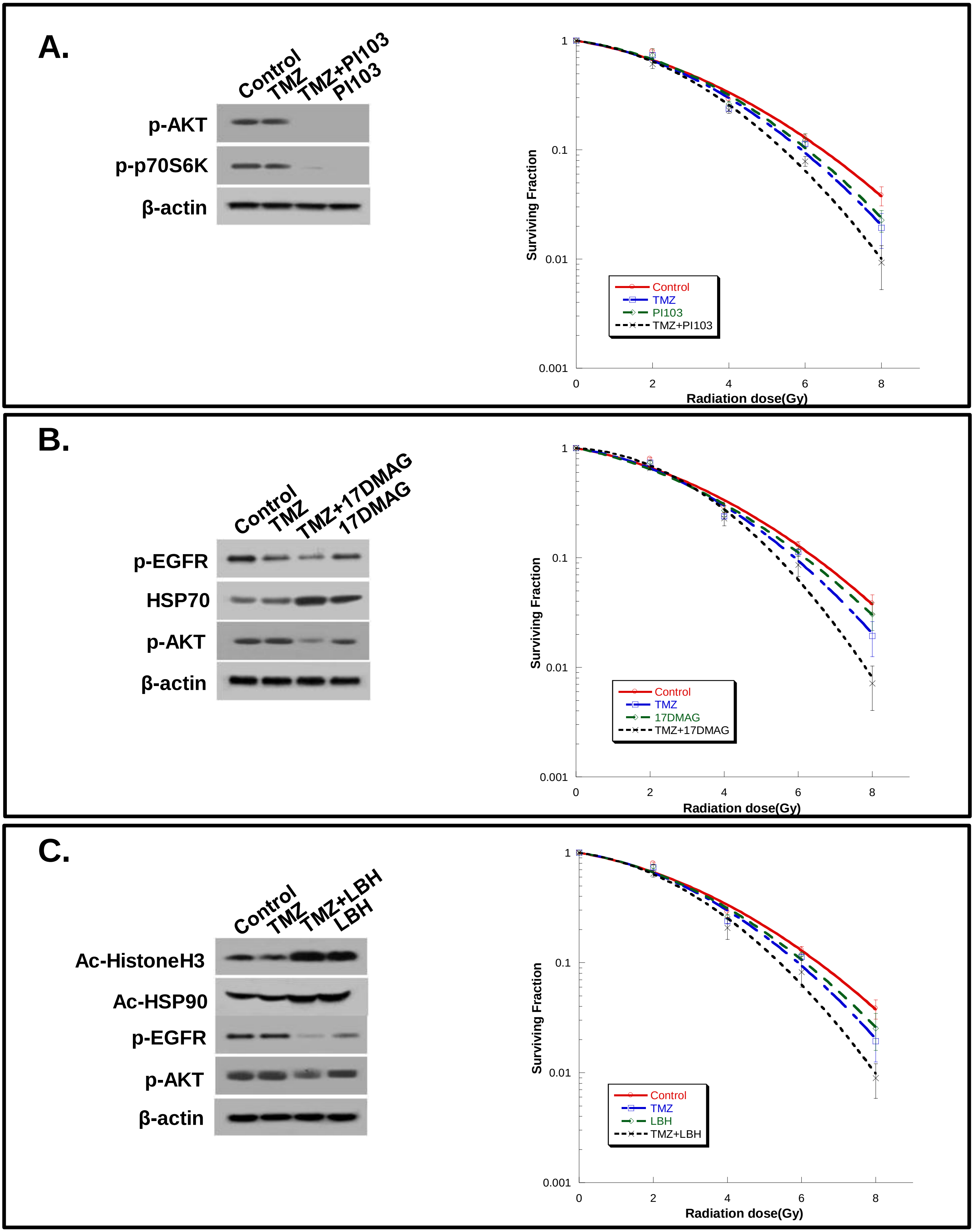

Supplemental data Fig.S2

A.

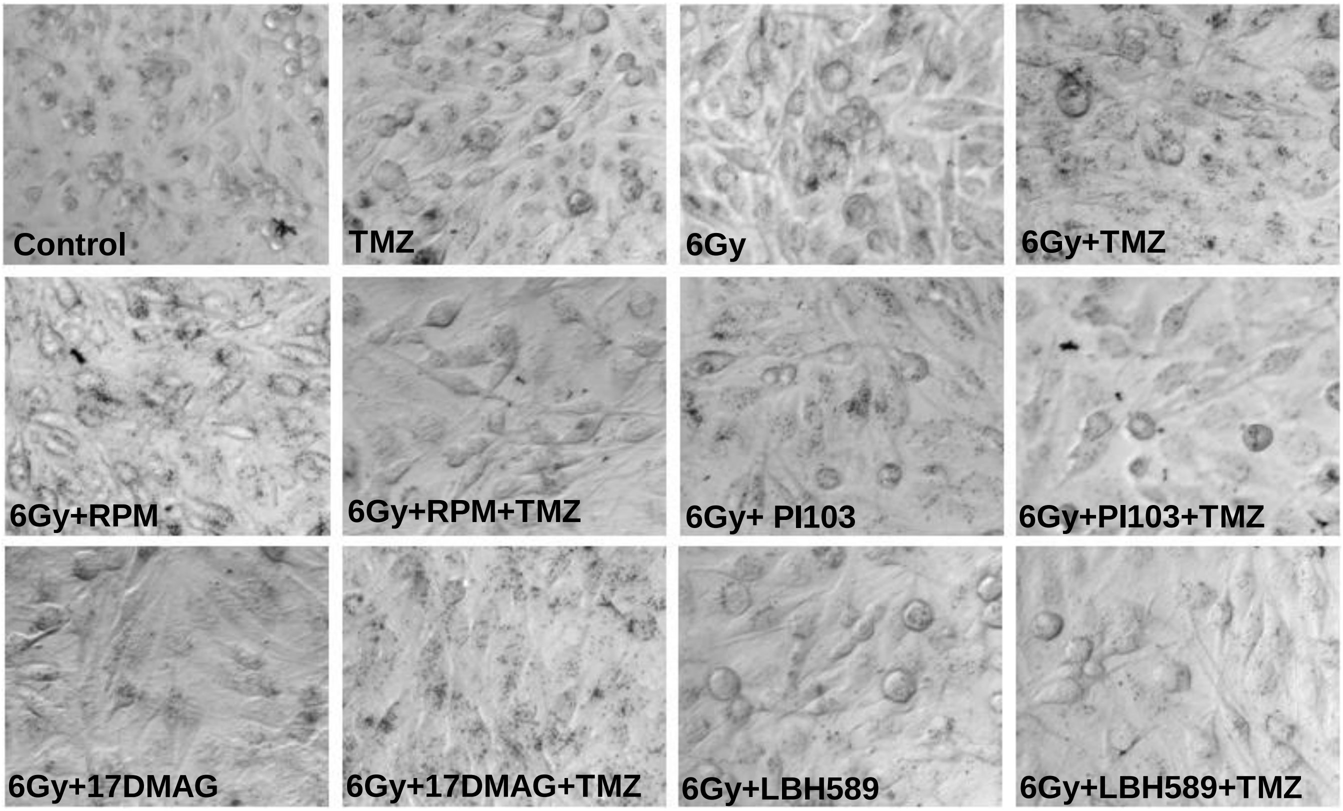

Supplemental data Fig.S3

A.

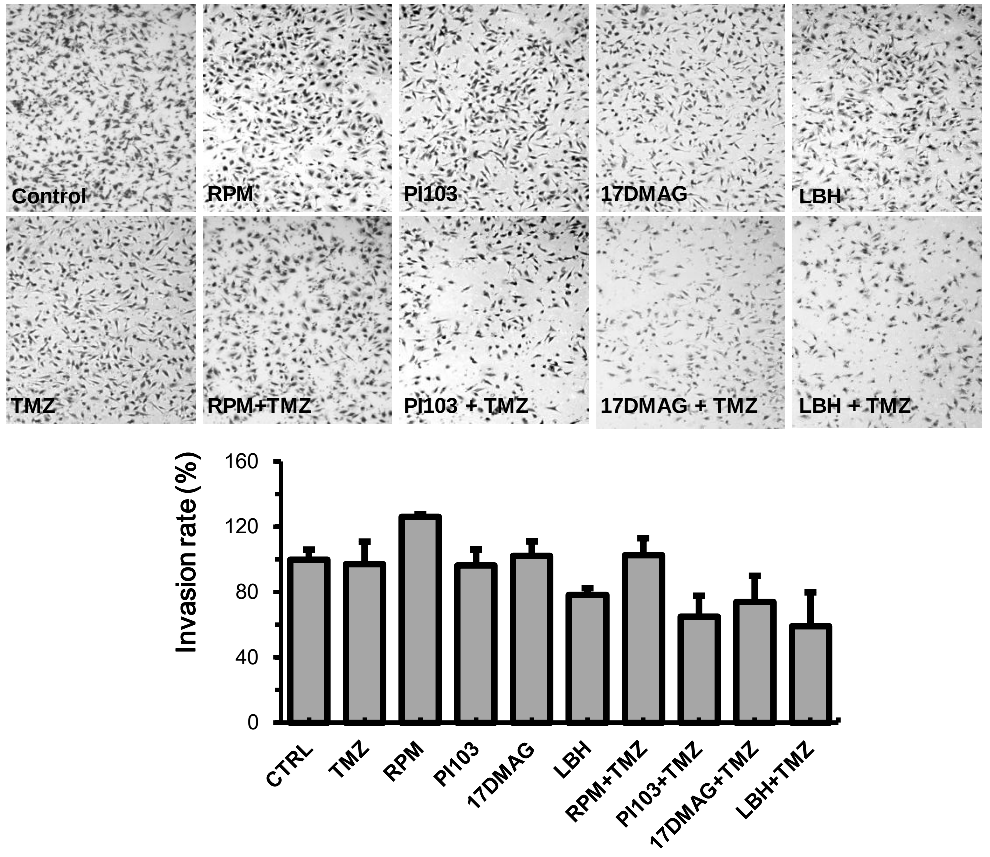

B.

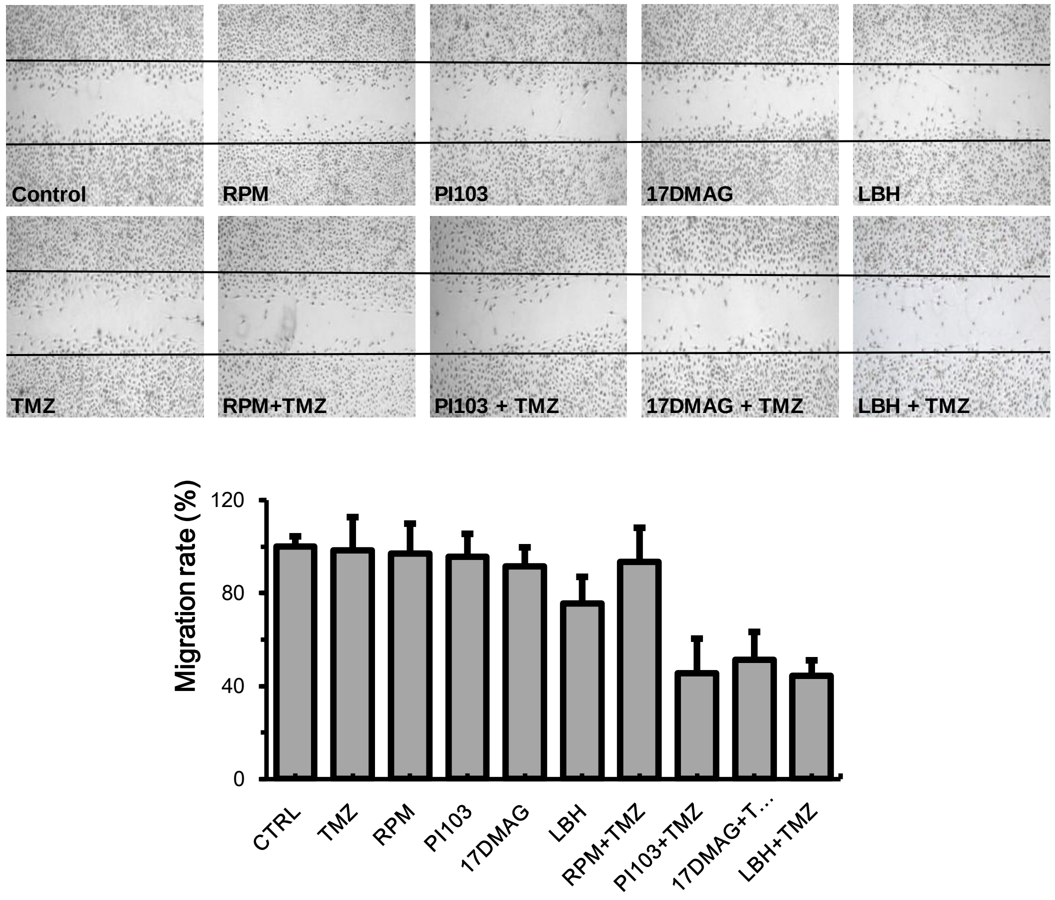

Supplement: Additional file 1: Table S1 — Sensitizer enhancement ratio of U251 cells. Table S2. Sensitizer enhancement ratio of T98G cells. Table S3. Sensitizer enhancement ratio of U87 cells. Figure S1. Clonogenic survival of U87 glioma cells after each treatment. (A) The effects of PI103 and TMZ on the radiosensitivity of U87MG. (B) The effects of 17DMAG and TMZ on the radiosensitivity of U87MG. (C) The effects of LBH589 and TMZ on the radiosensitivity of U87MG. Each experiment was repeated three times with similar results. Figure S2. Cellular Senescence-Associatedβ-Galactosidase Assay in U251 glioma cells after each treatment. Figure S3. Invasion, migration and vasculogenic mimicry formation of U251 glioma cells (without radiation). [file 1471-2407-14-17-S1.pdf]
